# Phenotypic variation of dairy cows’ hematic metabolites and feasibility of non-invasive monitoring of the metabolic status in the transition period

**DOI:** 10.3389/fvets.2024.1437352

**Published:** 2024-11-25

**Authors:** Silvia Magro, Angela Costa, Damiano Cavallini, Elena Chiarin, Massimo De Marchi

**Affiliations:** ^1^Department of Agronomy, Food, Natural Resources, Animals and Environment, University of Padova, Legnaro, Italy; ^2^Department of Veterinary Medical Sciences, Alma Mater Studiorum University of Bologna, Bologna, Italy

**Keywords:** metabolite, ketosis, negative energy balance, mid-infrared spectroscopy, reference interval

## Abstract

**Introduction:**

The incidence of metabolic diseases tends to be highest during the transition period (±3 weeks around parturition) in dairy cows due to physiological changes and the onset of lactation. Although blood profile testing allows for the monitoring of nutritional and metabolic status, conducting extensive analyses in the herd is costly and stressful for cows due to invasive procedures. Therefore, mid-infrared spectroscopy (MIR) could be seen as a valid alternative.

**Methods:**

In the present study, we used laboratory-determined reference blood data and milk spectra of 349 Holstein cows to (i) identify the non-genetic factors affecting the variability of major blood traits in healthy cows and, subsequently, (ii) test the predictive ability of milk MIR. Cows belonged to 14 Italian commercial farms and were sampled once between 5 and 38 days in milk. For *β*-hydroxybutyrate (BHB), non-esterified fatty acids (NEFA), cholesterol, glucose, urea, total protein, albumin, globulin, minerals, aspartate aminotransferase, gamma-glutamyl transferase, creatine kinase, total bilirubin, and cortisol, the effects of parity, days in milk, and season were investigated using a linear model.

**Results and discussion:**

The results indicate that all fixed effects significantly affected the hematic concentration of most of the traits. Regarding MIR, the most predictable traits were BHB, NEFA, and urea, with coefficients of determination equal to 0.57, 0.62, and 0.89, respectively. These values suggest that MIR predictions of BHB and NEFA are not sufficiently accurate for precise and punctual determination of the hematic concentration, however, still the spectrum of the milk can be exploited to identify cows at risk of negative energy balance and subclinical ketosis. Finally, the predictions can be useful for herd screening, decision-making, and genetic evaluation.

## 1 Introduction

The transition period conventionally refers to ±3 weeks around parturition and is known to be a challenging moment for dairy cows. In this phase the incidence of infections and metabolic diseases tends to be high due to nutritional, metabolic, hormonal, and immunological changes (1, 2) and all disorders/diseases that occur have severe implications on the cow’s productive performance and fertility during the lactation. This is because dairy cows commonly experience a negative energy balance (NEB), a condition caused by insufficient dry matter intake. Energy intake fails to meet the demands of both maintenance and milk production (3, 4).

To compensate for the lack of energy substrates, there is mobilization of body reserves that leads to an increase in the concentrations of NEB blood biomarkers such as non-esterified fatty acids (NEFA) and ketone bodies such as *β*-hydroxybutyrate (BHB) (2, 5).

Apart from the indicators of NEB, there are several blood traits important for dairy cow health monitoring, namely albumin, globulin, urea, hepatic aspartate aminotransferase (AST), gamma glutamyl transferase (GGT), creatine kinase (CK), and total bilirubin (BILT). Some of these parameters can vary simoultaneously, especially in some specific moments and/or in unhealthy animals. For instance, cows with metritis exhibit greater AST and BILT than healthy cows (6), and CK and AST concentrations jointly increase in uterine tissue in the presence of clinical endometritis (7).

Blood metabolic profile testing is time-consuming, invasive, and relatively expensive. Therefore, conducting extensive analyses on a large scale is not feasible in the field, also due to the absence of standardized and validated sampling protocols and analyses (8).

In this context, there is an evident interest in the Fourier-transform mid-infrared spectroscopy (FT-MIR), whose spectral data – already adopted to assess milk composition assessment (9, 10) – can be exploited to predict blood parameters (11, 12). Predicting mineral and hepato- and protein-profile indicators from milk spectra can represent a concrete and helpful opportunity for commercial farms. The present study aimed to (i) investigate the factors determining the variability of blood traits in healthy Holstein cows in early lactation, (ii) evaluate the association between blood and milk traits, and (iii) test the predictive ability of milk FT-MIR to predict hematic metabolic indicators.

## 2 Materials and methods

### 2.1 Sample collection

For this study, 14 commercial farms located in the Veneto region (Italy) were enrolled. The focus of this study was on Holstein cows under an intensive farming system. This means that the involved farms were characterized by a free stall barn, total mixed ration feeding, and no access to pasture. Between May and December 2020, each farm was visited once to sample cows in the first stage of lactation, i.e., between 3 and 38 days in milk (DIM). Animals with clinical signs of disease or those who received medical treatment at calving were excluded *a priori*, resulting in 349 clinically healthy cows, from calving up to the day of milk/blood sampling.

**Figure 1:**
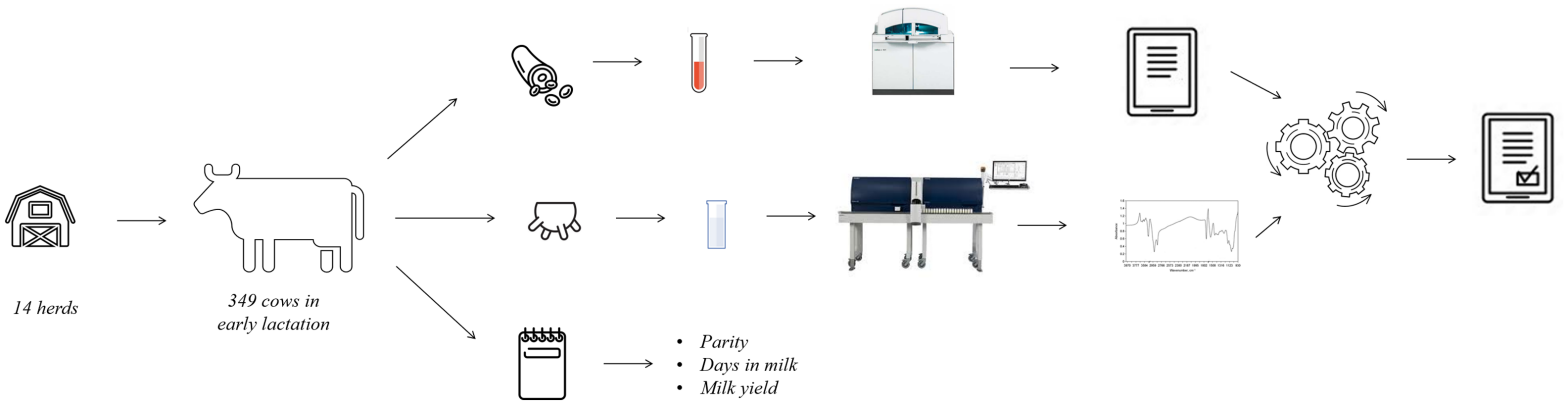
Experimental design and rationale of the study.

Individual blood (8 mL) and milk samples (50 mL) were collected as represented in Figure 1 during the morning milking, and information on DIM, parity, and milk yield (kg/d) was retrieved. Milk sampling was done in correspondence of the monthly official milk testing, and the tubes contained Bronopol (2-bromo 2-nitro 1,3-propandiol) for preservation in line with the guidelines of the International Committee for Animal Recording (ICAR). Milk samples were transported to the milk laboratory of the Breeders Association of Veneto Region (ARAV, Vicenza, Italy) for FT-MIR spectrum acquisition.

Blood sampling via venepuncture took place immediately after milking (within 1 h maximum, Figure 1), as performed in other studies (11, 13), and required immobilization of the animals in a containment trunk and preliminary cleaning of the venipuncture site (tail) with a disposable paper towel. Blood samples were stored into vacuum tubes containing lithium heparin (Greiner Bio-One GmbH, Kremsmünster, Austria) and were gently inverted 5 times, stored at 5°C and transported to the laboratory of the Experimental Zooprophylactic Institute of the Venezie (IZSVe, Padova, Italy).

### 2.2 Laboratory analysis

At the ARAV milk laboratory, samples (1 per cow) were scanned with a CombiFoss™ 7 machine (Foss Electric A/S, Hillerød, Denmark). For all cows, fat, protein, casein, and lactose content, and BHB and urea concentrations were determined via FT-MIR spectroscopy and somatic cell count (SCC, cells/mL) via flow cytometry. Infrared spectroscopy involves passing electromagnetic radiation through matter and measuring the energy absorbed at each wavelength (*n* = 1,060 in the case of CombiFoss™ 7). FT-MIR is commonly used in official milk laboratories to determine traditional milk quality traits for Dairy Herd Improvement as per ICAR guidelines (14). In this study, spectral information containing 1,060 infrared transmittance data points in the region between 5,000 and 900 cm^−1^ was stored using MilkoScan ™ 7 RM (Foss Electric A/S, Hillerød, Denmark).

At the IZSVe laboratory, blood samples (1 per cow) were centrifuged at 1,500 x g for 15 min at 4°C to separate the plasma. A set of parameters routinely used for blood profiling of lactating cows was considered: parameters related to both protein and energy metabolism (total protein, globulin, albumin, urea, glucose, NEFA, BHB, and cholesterol), enzymes and hepatic markers (AST, GGT, CK, and BILT), minerals (Ca, P, Mg, Na, K, and Cl) and cortisol. All plasma samples were analyzed with the COBAS C501 biochemical analyzer (Roche Diagnostics GmbH, Mannheim, Germany) using Roche BM commercial kits, except for the concentrations of NEFA and BHB, which were measured with an enzymatic colorimetric method (Randox Laboratories Ltd., Ardmore, United Kingdom). The analytical methods used for each blood parameter have been widely described by Cozzi et al. (15) who analyzed blood samples in the same laboratory. Intra-assay variation ranged from 0.31% for Cl to 4.17% for BILT. The inter-assay variation ranged from 0.47% for Na to 5.46% for BILT.

A database of blood parameters, milk spectral data, and individual cow information was created for subsequent statistical analyses.

### 2.3 Phenotypic variance

The software R v. 4.1.2 (16) was used for data handling and calculation of descriptive statistics and correlations. Traits were visually inspected to evaluate the Gaussian distribution, and whenever necessary, values underwent transformation to achieve normality. In particular, the values of BHB, NEFA, AST, GGT, CK, and cortisol were log_10_ transformed.

Values of plasma and milk traits deviating more than 3 SD from the respective mean were treated as missing deviating more than 3 SD from the respective mean were treated as missing. Spearman’s correlations were calculated between plasma traits and between plasma and milk traits.

To investigate the factors affecting plasma traits, analysis of variance was performed using the following generalized linear mixed model (Equation 1) with PROC GLIMMIX in SAS software v. 9.4 (SAS Institute Inc., Cary, NC, United States):


(1) $$y_{\text{ijklm}}=\mu +P_{i}+D_{j}+{\left(P\;x\;D\right)}_{ij}+M_{k}+H_{m}+e_{\text{ijklm}}$$

where *y_ijklm_* is the trait investigated; *μ* is the overall intercept of the model; *P_i_* is the fixed effect of the *i*th parity (*i* = 1, 2, ≥3, with the last class including cows up to parity 10); *D_l_* is the fixed effect of the *l*th distance from calving (*l* = 4 classes, with the first being a class from 3 to 8 DIM, followed by 2 classes of 6 d each, and the last being a class from 21 to 38 DIM); (*P x D*) is the first order interaction between parity and DIM class; *M_k_* is the fixed effect of the *k*th sampling season (*k* = 3 classes, May-Jun, Jul-Aug, Sep-Oct); *H_m_* is the random effect of the *m*th herd (*m* = 14 levels) assumed to be distributed as ~N(0, σ^2^_H_), where σ^2^_H_ is the herd variance; and *e_ijklm_* is the random residual assumed to be distributed as ~N(0, σ^2^_e_), where σ^2^_e_ is the residual variance. Multiple comparisons of least squares means (LSM) were performed using Bonferroni adjustment with significance at *p* ≤ 0.05. The results for log-transformed variables were reported after back transformation.

Blood BILT was processed as a binary trait, with a value of ‘1’ assigned if BILT was >4 μmol/L and ‘0’ if it was ≤4 μmol/L. This was done because several samples presented BILT concentration below the laboratory limit of detection, i.e., < 2.5 μmol/L. A binary logistic regression analysis was performed using the model above described (Equation 1) in SAS v. 9.4. For each fixed effect, odds ratios were calculated and were considered as significant when the 95% confident interval did not include 1.

### 2.4 Mid-infrared prediction models

Each milk spectrum was paired with the relative blood sample values of BHB, NEFA, glucose, cholesterol, urea, Ca, P, Mg, Na, K, Cl, total protein, albumin, globulin, GGT, AST, CK, and cholesterol determined in the laboratory. For non-distributed traits, a logarithmic transformation was also applied. For each spectral wavelength, the value was transformed from transmittance to absorbance using the formula: Absorbance = log_10_(1/Transmittance).

Spectral regions where there is non-negligible noise due to water absorption were discarded (10, 17), leading to 338 wavelengths located/scattered in the following intervals within the spectrum: 945.5 to 1,585.6 cm^−1^, 1,716.8 to 1,929.0 cm^−1^, 2,507.7 to 2,970.7 cm^−1^. Preliminary analysis of the spectral data was conducted using principal component analysis to identify anomalous samples in terms of the FT-MIR spectrum. This allowed for the elimination of nine spectral outliers. Before the development of the prediction model, spectra were independently subjected to standard normal variate (SNV) scatter correction with the aim of normalizing the spectral data to reduce baseline shifting or tilting due to nonspecific radiation scattering (18). The partial least squares (PLS) analysis was carried out using the ‘trainControl’ function available in the R package ‘caret’, whose details are described in Kuhn (19), following this process:

- The models were fine-tuned using leave-one-out cross-validation, and the number of components was set automatically but capped at a maximum of 20 to avoid overfitting;
- Spectral data points were centered and scaled;
- Predictions with residuals largely different from the observed values were removed;
- The complete data set was randomly split into a calibration set representing 70% of the total samples and a validation set (30%), both of which had similar mean and standard deviation of the target trait and were representative of all herds, parities, and DIM;
- The external validation was iterated 3 times, each time over a different portion of cows, and the reported fitting statistics were the average of the fitting statistics were the average of the fitting statistics of the 3 iterations.

The fitting statistics of the PLS consisted of: the standard error in leave-one-out cross-validation and external validation and the coefficient of determination (R^2^) in leave-one-out cross-validation and external validation.

Using existing thresholds of BILT, BHB, and NEFA (20), partial least squares discriminant analysis (PLS-DA) was used through the same package to classify animals above or below the thresholds (e.g., 4 μmol/L for BILT), and therefore potentially at risk of certain metabolic disorders. PLS-DA performance included sensitivity, specificity, positively predictive values, negatively predictive values, and balanced accuracy in both calibration and validation. Balanced accuracy is the mean of sensitivity and specificity, and positively and negatively predictive values are the proportions of positive and negative results that are true positive and true negative, respectively.

## 3 Results and discussion

### 3.1 Plasma traits

#### 3.1.1 General overview

There is a metabolic priority toward the mammary gland in dairy breeds, so energy and nutrients primarily support milk synthesis. As reported by Bauman and Currie (21), in fact, “[…] the most pronounced example of homeorhesis would be in a dairy cow where initiation of lactation dramatically alters metabolism of many maternal organs in order that the mammary gland be supplied with nutrients necessary for synthesis of milk […].” The genetic pressure that dairy cattle breeds were exposed to in the past resulted in a progressive linear increase in milk productivity at the expense of fitness, fertility, resistance to disease, and overall health (22). This explains why conditions such as NEB, glucose drop and hyperketonemia are commonly considered normal and are observed in healthy animals with no clinical signs.

Descriptive statistics of the milk and plasma traits analyzed for early lactation cows before editing are presented in Tables 1, 2, respectively. The cows sampled had an average and SD of DIM and parity of 14.54 ± 7.62 and 2.40 ± 11.41, respectively. The average milk composition was in line with the average performance reported by the Italian Breeders Association (AIA) for Holsteins farmed in the Veneto region in 2020 (23).

**Table 1:** Descriptive statistics of milk traits before editing^1^.

Milk trait^2^	Mean (SD)	Minimum	Maximum	N. missing values^3^	
				< Mean-3 SD	< Mean-3 SD
Yield, kg/d	39.04 (10.47)	10.00	62.80	0	1
Fat, %	4.39 (1.39)	0.67	13.08	0	2
Protein, %	3.34 (0.49)	2.36	6.59	0	4
Casein, %	2.57 (0.39)	1.72	5.22	0	4
Lactose, %	4.73 (0.26)	3.58	5.28	0	6
SCS	2.62 (2.09)	−1.64	9.38	0	0
Urea, mg/dL	27.39 (7.85)	10.8	65.6	0	2
Log_10_ BHB	−0.99 (0.24)	−3.00	−0.24	0	5

^1^Values of milk traits deviating more than 3 SD from the respective mean (if requested with log-transformed values) were treated as missing. ^2^SCS, somatic cell score; calculated as SCS, 3 + log2 (SCC/100,000), where SCC is somatic cell count (cells/mL); BHB, β-hydroxybutyrate (mmol/L).

**Table 2:** Descriptive statistics of blood traits before editing^1^.

Blood trait^2^	Mean (SD)	Minimum	Maximum	N. missing values^3^	
				<Mean-3 SD	> Mean + 3 SD
Energy profile					
Glucose, mmol/L	2.80 (0.53)	0.20	4.90	2	1
Cholesterol, mmol/L	2.66 (0.92)	0.60	6.00	0	2
NEFA, mmol/L	0.52 (0.34)	0.04	2.02	0	6
BHB, mmol/L	0.71 (0.54)	0.15	4.96	0	10
Urea, mmol/L	4.19 (1.31)	1.30	9.30	0	2
Mineral profile					
Na, mmol/L	138.88 (3.49)	129.00	150.00	0	1
K, mmol/L	4.39 (0.39)	2.61	5.94	1	3
Cl, mmol/L	98.54 (3.77)	71.00	111.00	1	2
Ca, mmol/L	2.45 (0.15)	1.98	3.21	1	2
P, mmol/L	1.60 (0.34)	0.63	3.11	0	3
Mg, mmol/L	0.96 (0.13)	0.56	1.40	2	1
Protein profile					
Total protein, g/L	74.37 (6.11)	59.00	98.00	0	1
Albumin, g/L	33.91 (4.34)	17.00	43.00	1	0
Globulin, g/L	40.46 (7.05)	26.00	66.00	0	6
Hepatic and muscular profile					
AST, U/L	98.14 (47.97)	53.00	629.00	0	3
GGT, U/L	16.30 (9.74)	4.00	152.00	0	2
CK, U/L	340.34 (694.34)	79.00	6,515.00	0	7
Stress profile					
Cortisol, nmol/L	17.05 (15.00)	1.94	107.00	0	4

^1^Values of blood traits deviating more than 3 SD from the respective mean were treated as missing. ^2^NEFA, not esterified fatty acids (mmol/L); BHB, β-hydroxybutyrate (mmol/L); AST, aspartate aminotransferase (U/L); GGT, gamma glutamyl transferase (U/L); CK, creatin kinase (U/L). ^3^Excluded for the analysis of variance. In the case of non-normally distributed traits, the missing values were identified using the log-transformed form (BHB, NEFA, AST, GGT, CK and cortisol).

Hematic traits are in the range of values commonly found in healthy high-producing cows (24). The minimum and the maximum values of BHB in this study were quite far from each other, 0.15 and 4.96 mmol/L, suggesting that the healthy cows involved differ widely in terms of mobilization.

In this study, the average NEFA was in line (0.52 mmol/L) with another study reporting the trend of this trait from 0 to 40 DIM (25). Blood urea, which is directly related to BUN, was within the conventional range (Table 2) in this study, except for a few cases presenting either a lower (< 1.70 mmol/L, 6 cows) or higher concentration (> 6.87 mmol/L, 11 cows).

Regarding the mineral profiles, the averages in Table 2 mirror those reported by Walter et al. (25). Due to osmotic equilibrium, Ca and P are expected to be scarcely variable in the blood of healthy cows. In the present study, it appeared that 6 cows out of 349 cows were in a state of hypocalcemia with a concentration of Ca below the threshold (2.10 mmol/L, 26). Total protein, albumin, and globulin were included in the protein profile (Table 2). Globulins are produced in response to inflammation of the chronic type and albumin is a protein that is synthesized in the liver and low levels reflect poor liver health or a poor supply of amino acids from the diet (27). The averages observed in the present study are in line with those of Cattaneo et al. (27), who evaluated the evolution of albumin and globulin concentrations before and after calving.

**Figure 2:**
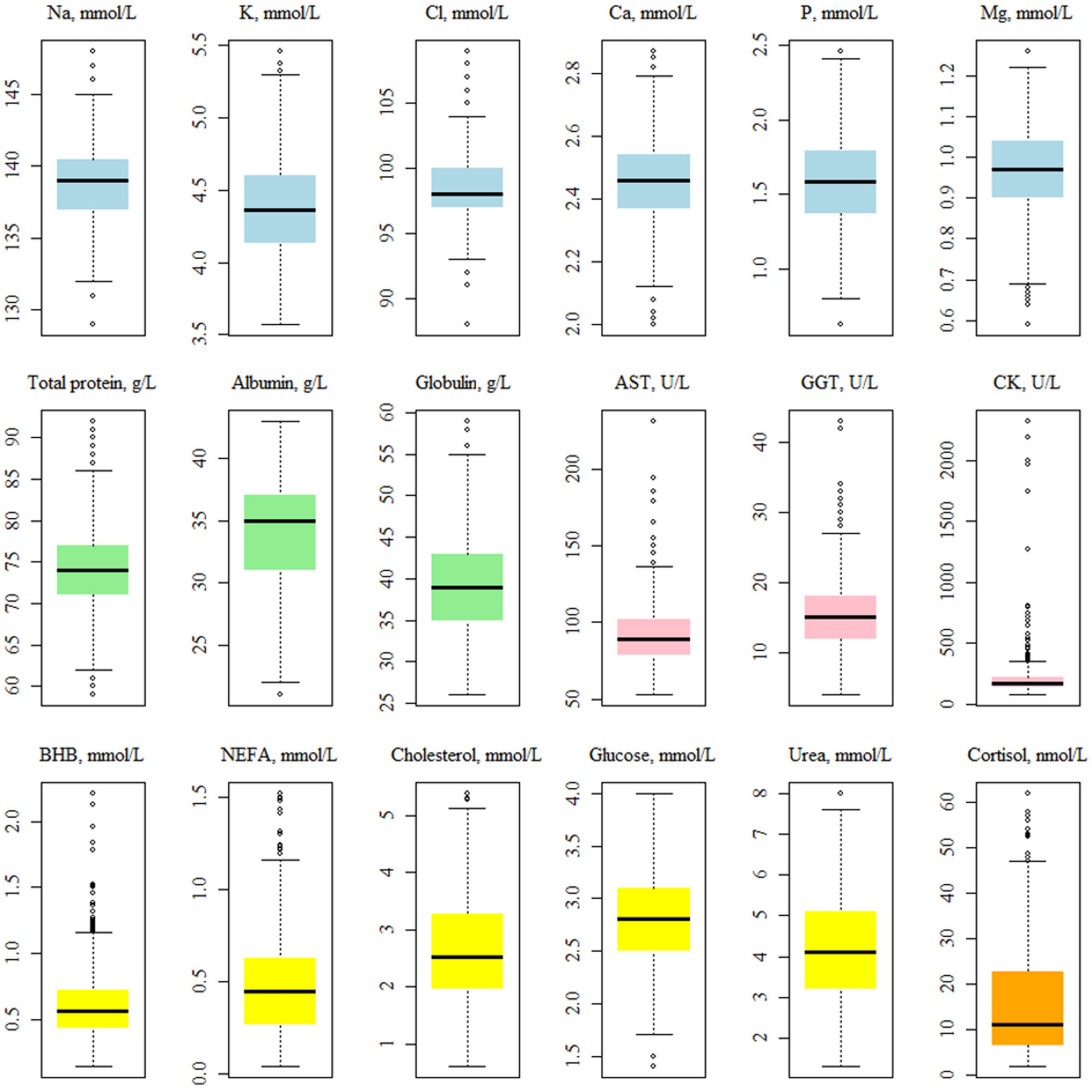
Boxplots showing the distribution^1^ of the plasma traits^2^ values. ^1^Before the log-transformation of the non-normally distributed traits, i.e., AST, GGT, CK, BHB, NEFA and cortisol. ^2^NEFA, not esterified fatty acids (mmol/L); BHB, *β*-hydroxybutyrate (mmol/L); AST, aspartate aminotransferase (U/L); GGT, gamma glutamyl transferase (U/L); CK, creatin kinase (U/L).

The average concentration of CK was 340.34 U/L (Table 2), while the median was 168 U/L. Walter et al. (25) indicated that the distribution of CK values is not normal; thus, the mean can be inflated by a limited number of samples (Figure 2). The average of both AST and GGT levels was in accordance with Fiore et al. (28), who evaluated how blood parameters were affected by different preventive treatments for hyperketonemia.

Cortisol is a valid indicator of chronic stress in livestock animals and, is detectable in a variety of matrices, e.g., saliva, milk, plasma, and hair (29). In this study, the minimum, average, and maximum values observed for this hormone were 1.94, 17.05, and 107.00 nmol/L.

#### 3.1.2 Correlations

**Figure 3:**
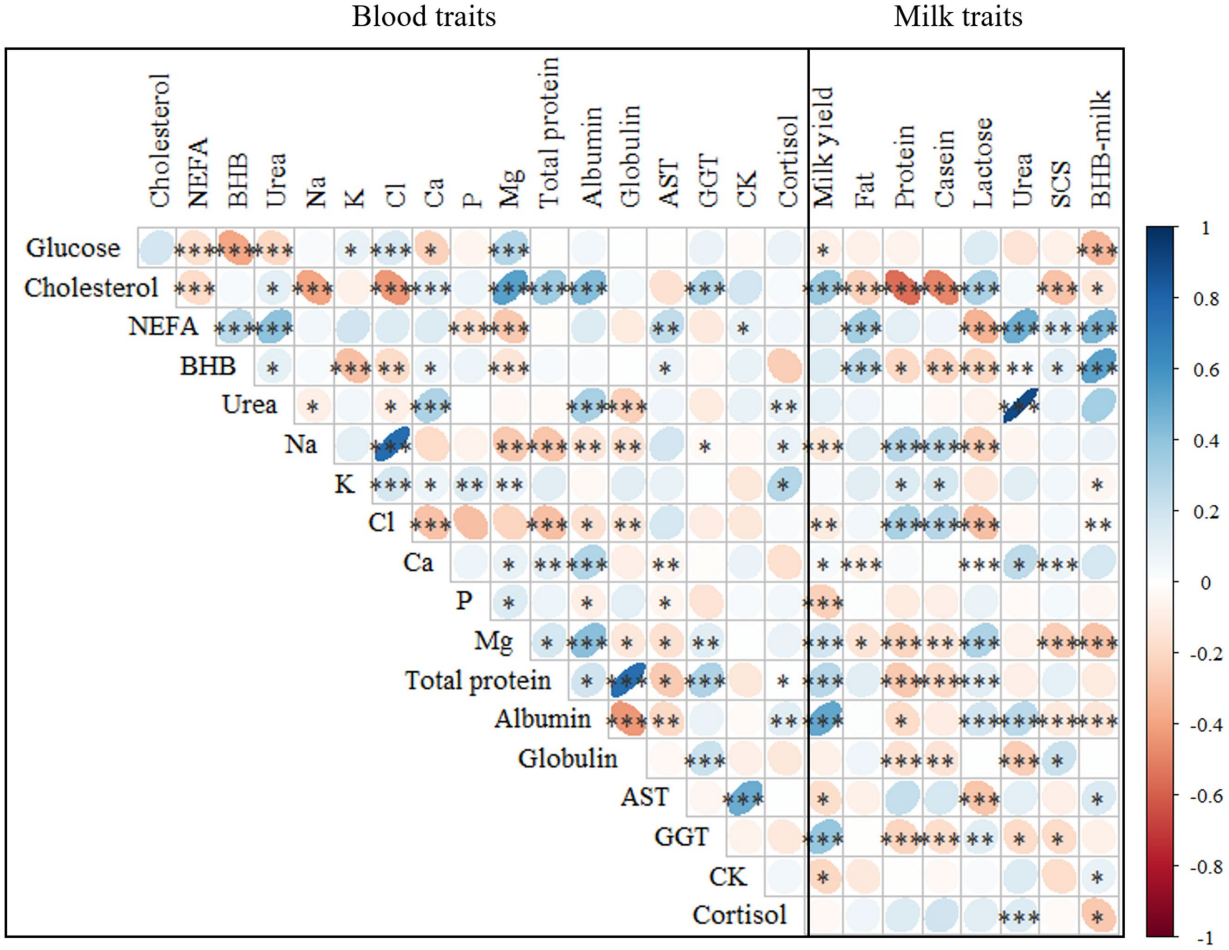
Spearman’s correlations^1^ between the investigated blood and milk traits^2^. ^1^***p ≤ 0.001; **p ≤ 0.01; *p ≤ 0.05. ^2^NEFA, not esterified fatty acids (mmol/L); BHB, β-hydroxybutyrate (mmol/L); AST, aspartate aminotransferase (U/L); GGT, gamma glutamyl transferase (U/L); CK, creatin kinase (U/L); SCS, somatic cell score, calculated as SCS, 3 + log_2_(SCC/100,000), where SCC is somatic cell count (cells/mL).

Spearman’s correlations were calculated to evaluate if an association exists among blood parameters and between blood and milk traits (Figure 3). Such an investigation is useful for understanding how milk is capable of mirroring blood and, in addition, for determining whether some hematic traits change in parallel or not in the transition period.

Among the energy profile traits, the strongest positive and negative correlations were found between NEFA and urea (0.40) and between BHB and glucose (−0.39), respectively (Figure 3). A significant association between hypoglycemia, fat mobilization, and circulating nitrogen has been reported in previous studies that reported blood traits in cows within 35 DIM (11, 12). On average, these traits showed weak correlations with mineral and protein profiles. In particular, urea was positively correlated with albumin (0.40) and negatively with globulin (−0.24), as reported by Luke et al. (12). Among the mineral profile traits, the strongest positive and negative correlations were found between Na and Cl (0.76) and between Cl and Ca (−0.29). The traits in the protein profile set were negatively correlated with Na and Cl. Moreover, in line with Luke et al. (12), a positive and moderate correlation was observed between albumin and Ca and Mg. Among the hepatic and muscular profile traits, the strongest correlation was found between CK and AST (0.49), and these traits showed weak correlations with NEFA and BHB (Figure 3). In addition, Sattler and Fürll (7) reported a positive correlation between CK and AST, both of which increased in the presence of uterine diseases.

According to the literature, blood NEFA, BHB, and urea are expected to go in parallel, especially in cows with severe NEB. However, their association could be nonlinear or weak (30). For instance, McCarthy et al. (31) found that the correlation between BHB and NEFA in the postpartum is moderate, despite positive (0.26).

Correlations were also calculated between plasma and milk traits (Figure 3). The strongest correlation was observed between urea in plasma and milk (0.89), followed by BHB (0.53). In agreement with Benedet et al. (11), milk BHB was positively correlated with NEFA and negatively correlated with glucose, while cholesterol was inversely associated with milk quality (i.e., fat, protein, and casein content). Blood BHB was positively correlated with fat content while negatively with protein, casein and lactose content. This association has also been reported by Benedet et al. (11). Regarding milk yield, the strongest positive and negative correlations were found with albumin (0.51) and P (−0.24), respectively, whereas, SCS was negatively correlated with Mg and cholesterol. On the other hand, Andjelić et al. (32) observed a negative correlation between milk fat and NEFA and BHB. Andjelić et al. (32) reported a positive correlation between blood and milk serum AST and GGT levels.

#### 3.1.3 Variation across DIM

The transition from the dry-off to lactogenesis in the first days after calving is critical for dairy cow. After calving, especially if the diet is not properly balanced in the *pre-partum* period, nutritional status can play a dramatic role. For example, a significant reduction in hematic Ca and P is frequently observed around or immediately after calving, which can expose cows to hypocalcemia and other associated disorders (33). Furthermore, during this period, dairy cows undergo a natural state of immunosuppression, which is often considered the principal cause of their high vulnerability to infectious diseases such as mastitis or metritis (34). Monitoring changes in blood biomarkers can be informative, and the early identification of subclinical metabolic disorders in the first days after calving is advisable and helpful, allowing for the detection of anomalies, animals at risk, proper decision-making, and timely management interventions (35).

The LSM of DIM classes are presented in Table 3 and revealed that NEFA concentration was lower in 21–38 DIM compared to the classes of DIM closest to calving (i.e., 3–8 DIM and 9–14 DIM), and that BHB concentration was numerically greater in the first two DIM classes (i.e., 3–8 DIM and 9–14 DIM) than the other two (i.e., 15–20 DIM and ≥ 21 DIM, Table 3). An increase in NEFA is expected in dairy cows in the first days after calving and is commonly observed in high-producing breeds, due to a massive mobilization of adipose reserves in dairy cows to overcome NEB (5). In contrast, the LSM of glucose concentration did not fluctuate dramatically in the first 38 DIM in the present study. Although there may be peaks or drops in glucose in correspondence of meals, the baseline level is subject to tight homeostatic regulation; therefore, glycemia is considered informative in animals suffering from specific disorders/pathologies (36). Cholesterol concentration increased with DIM, progressively raising from the first class onward (Table 3), likely due to its relationship with the dry matter intake of cows, which increases in the first week after calving (37). This finding was also observed by Walter et al. (25) in 362,586 serum samples obtained from clinically healthy German Holstein cows.

**Table 3:** Least squares means (standard error) of blood traits^1^ for the fixed effect of days in milk/ after calving (DIM) along with the *F*-value.

Blood trait	3–8 DIM	9–14 DIM	15–20 DIM	≥21 DIM	F-value
Energy profile					
Glucose, mmol/L	2.91 (0.10)	2.79 (0.10)	2.88 (0.10)	2.92 (0.10)	*ns*
Cholesterol, mmol/L	2.05^d^ (0.15)	2.45^c^ (0.15)	2.90^b^ (0.15)	3.49^a^ (0.15)	69.40 ***
NEFA, mmol/L	0.42^a^ (0.06)	0.41^a^ (0.06)	0.35^ab^ (0.05)	0.29^b^ (0.04)	7.15 ***
BHB, mmol/L	0.61 (0.03)	0.62 (0.03)	0.57 (0.03)	0.59 (0.03)	*ns*
Urea, mmol/L	3.60 (0.30)	3.62 (0.30)	3.52 (0.30)	3.77 (0.30)	*ns*
Mineral profile					
Na, mmol/L	140.23^a^ (0.64)	139.27^ab^ (0.63)	138.67^b^ (0.63)	138.27^b^ (0.64)	8.08 ***
K, mmol/L	4.35 (0.07)	4.40 (0.07)	4.42 (0.07)	4.38 (0.07)	*ns*
Cl, mmol/L	100.03^a^ (0.63)	98.88^b^ (0.62)	98.37^b^ (0.62)	97.72^b^ (0.62)	11.82 ***
Ca, mmol/L	2.41^b^ (0.02)	2.46 ^ab^ (0.02)	2.43^ab^ (0.02)	2.48^a^ (0.02)	5.70 **
P, mmol/L	1.50^b^ (0.06)	1.57^ab^ (0.06)	1.63^ab^ (0.06)	1.67^a^ (0.06)	5.12 **
Mg, mmol/L	0.90^c^ (0.01)	0.96^b^ (0.01)	0.99^ab^ (0.01)	1.02^a^ (0.01)	18.13 ***
Protein profile					
Total protein, g/L	70.53^c^ (0.77)	72.70^b^ (0.74)	75.58^a^ (0.76)	76.82^a^ (0.78)	21.79 ***
Albumin, g/L	32.87^b^ (0.60)	32.96^b^ (0.58)	33.42^b^ (0.59)	35.58^a^ (0.60)	8.66 ***
Globulin, g/L	37.31^b^ (0.90)	39.54^a^ (0.86)	41.61^a^ (0.88)	40.93^a^ (0.90)	8.66 ***
Urea, mmol/L	3.60 (0.30)	3.62 (0.30)	3.52 (0.30)	3.77 (0.30)	*ns*
Hepatic and muscular profile					
AST, U/L	102.82^a^ (4.22)	100.05^a^ (4.09)	92.34^ab^ (4.00)	87.39^b^ (3.44)	4.87 *
GGT, U/L	13.67^b^ (0.52)	14.51^b^ (0.53)	17.31^a^ (0.70)	17.11^a^ (0.71)	8.75 ***
CK, U/L	265.04 (26.28)	217.91 (22.23)	220.43 (22.08)	197.60 (20.20)	*ns*
Stress profile					
Cortisol, nmol/L	12.50 (1.60)	13.41 (1.66)	16.51 (2.09)	14.56 (1.85)	*ns*

^1^NEFA, not esterified fatty acids (mmol/L); BHB, β-hydroxybutyrate (mmol/L); AST, aspartate aminotransferase (U/L); GGT, gamma glutamyl transferase (U/L); CK, creatin kinase (U/L). Estimates with different superscript letters within row are statistically significantly different (*p* ≤ 0.05).

The P, Ca, and Mg concentrations were the lowest immediately after calving (3–8 DIM), followed by a gradual increase (Table 3). Luke et al. (12) observed a similar trend for Ca and Mg concentration in 773 spring-calving Holstein cows. Immediately after parturition, there is a significant and immediate demand for Ca for milk production (38). This, coupled with the typically low dry matter intake, results in substantially low Ca mobilization and Ca deficiency that requires several days to be adjusted (33, 38). In contrast, Mg and P play important roles in Ca homeostasis (33). Moreover, these minerals (i.e., Ca, Mg, and P) are present in high amounts in transition milk, and mature milk (39, 40). However, Na and Cl concentrations were the highest immediately after calving (3–8 DIM) and then gradually decreased (Table 3). The concentration of Cl was very high around calving, as it is common to increase the anionic quota (Cl and S) in prepartum diets to enhance feed intake and indirectly reduce the risk of hypocalcemia (41).

Total protein concentration was the lowest in the first class of DIM. Specifically, the lowest globulin concentration was observed in the period between 3 and 8 DIM, whereas the albumin concentration was relatively consistent across the 21 days and then reached the highest value in the last class, DIM ≥21 (35.58 g/L; Table 3). Similar patterns were observed by Luke et al. (12) who observed concentrations of about 34 g/L in 24–35 DIM.

With regard to the hepatic profile, a significant decrease in AST was observed from the first DIM class to the last (Class 1:102.82 vs. Class 4: 87.39 g/L). The GGT concentration showed an opposite trend, being the lowest immediately after calving (3–8 DIM, 9–14 DIM; Table 3). On the other hand, Moretti et al. (42) reported that the concentration of GGT was lower in the first days after calving (3 ± 1 DIM) due to the production of colostrum and milk and low feed intake. Hoffman and Solter (43) reported the highest concentration of GGT activity in the kidney, pancreas, intestines, and mammary glands. In contrast, CK and cortisol were not significantly affected by distance from calving, so the LSM of the DIM classes were not statistically different (Table 3).

Table 4 contains the odds ratio estimated for the BILT classes (> and ≤ 4 μmol/L). Overall, there were no differences related to distance from calving in the first 14 days, but LSM indicated that the probability to observe high BILT concentration (> 4 μmol/L) decreased as DIM progressed. It is likely that, higher LSM for AST were estimated near calving because they reflect tissue damage in the uterine tissue (25) and/or degradation of muscle cells caused by mobilization of body reserves (7). Authors of other studies (25, 44) observed that the highest levels of BILT are found in the first week postpartum as a consequence of the lipolysis.

**Table 4:** Odds ratio and 95% confidence interval for total bilirubin (BILT) class^1^ for each fixed effect.

Effect	Level	Prevalence ^2^, %	Odds ratio	95% confidence interval
Days in milk/after calving	3–8 DIM	41.57	Reference	
	9–14 DIM	37.62	0.82	0.43–1.54
	15–20 DIM	23.08	0.37 **	0.17–0.77
	≥21 DIM	19.23	0.20 ***	0.08–0.48
Parity	1	20.00	Reference	
	2	27.18	1.57	0.79–3.11
	≥3	43.61	3.85 ***	2.00–7.37
Season	May–June	31.01	Reference	
	July–August	31.16	2.88	0.98–8.50
	September–October	31.65	2.68	0.80–9.01

^1“1” if BILT was > 4 μmol/L and “0” if ≤ 4 μmol/L. 2of BILT class = 1.^

#### 3.1.4 Variation related to the effect of parity

The parity of cows played a crucial role in the variability of the plasma traits investigated, except for Na, Cl, K, albumin, AST, and cortisol (Table 5). Within the energy profile traits, glucose and cholesterol concentrations decreased with parity, whereas NEFA and BHB increased (Table 5). This is in line with the literature and supports that multiparous cows have a higher prevalence of hyperketonemia than primiparous due to greater milk production, mobilization of body reserves, and metabolic stress related to the previous lactation (5).

**Table 5:** Least squares means (standard error) of blood traits^1^ for the fixed effect of parity class along with the *F*-value.

Blood trait	Parity 1	Parity 2	Parity ≥3	*F*-value
Energy profile				
Glucose, mmol/L	3.05^a^ (0.10)	2.75^b^ (0.10)	2.83^b^ (0.10)	19.86 ***
Cholesterol, mmol/L	2.80^a^ (0.14)	2.81^a^ (0.14)	2.56^b^ (0.14)	6.27 **
NEFA, mmol/L	0.33^b^ (0.05)	0.34^b^ (0.05)	0.42^a^ (0.06)	7.56 **
BHB, mmol/L	0.54^b^ (0.02)	0.62^ab^ (0.03)	0.64^a^ (0.02)	5.77 **
Urea, mmol/L	3.57 (0.30)	3.80 (0.30)	3.51 (0.29)	3.28 *
Mineral profile				
Na, mmol/L	139.16 (0.62)	139.32 (0.62)	138.84 (0.61)	*ns*
K, mmol/L	4.40 (0.07)	4.43 (0.07)	4.33 (0.07)	*ns*
Cl, mmol/L	98.63 (0.61)	99.12 (0.61)	98.49 (0.60)	*ns*
Ca, mmol/L	2.45^ab^ (0.02)	2.47^a^ (0.02)	2.42^b^ (0.02)	4.13 *
P, mmol/L	1.71^a^ (0.04)	1.59^b^ (0.04)	1.48^b^ (0.04)	18.69 ***
Mg, mmol/L	0.99^a^ (0.01)	0.97^a^ (0.01)	0.94^b^ (0.01)	7.29 ***
Protein profile				
Total protein, g/L	72.84^b^ (0.72)	72.60^b^ (0.72)	76.29^a^ (0.67)	17.96 ***
Albumin, g/L	33.73 (0.57)	33.72 (0.56)	33.66 (0.53)	*ns*
Globulin, g/L	38.69^b^ (0.84)	38.79^b^ (0.84)	42.08^a^ (0.79)	13.04 ***
Hepatic and muscular profile				
AST, U/L	93.29 (3.73)	96.99 (3.71)	96.12 (3.26)	*ns*
GGT, U/L	14.04^b^ (0.50)	15.91^ab^ (0.57)	16.89^a^ (0.52)	7.87 **
CK, U/L	286.68^a^ (27.03)	220.68^b^ (21.17)	177.55^b^ (15.73)	16.00 ***
Stress profile				
Cortisol, nmol/L	15.21 (1.87)	13.95 (1.69)	13.40 (1.55)	*ns*

^1^NEFA, not esterified fatty acids (mmol/L); BHB, β-hydroxybutyrate (mmol/L); AST, aspartate aminotransferase (U/L); GGT, gamma glutamyl transferase (U/L); CK, creatin kinase (U/L). Estimates with different superscript letters within row are statistically significantly different (*p* ≤ 0.05).

As regards the mineral profile, Ca, P and Mg concentrations were the greatest in the first two parity orders (Table 5). In older cows, low minerals concentration can be associated with a decreased capacity to mobilize Ca from the bones, lower absorption at the intestinal level, and high requirement for Ca (33, 40).

In agreement with Cozzi et al. (15), total protein and globulin concentrations were greater in parity greater than 2 (Table 5) and GGT increased from parity 1 onwards (Table 5). On the other hand, the concentration of the CK was the greatest in parity 1 (Table 5). Cozzi et al. (15) and Walter et al. (25) hypothesized that the high CK level - a specific marker of skeletal muscle injury - in primiparous cows may be due to physical stress caused by hierarchical conflicts when they are grouped with multiparous cows.

Regarding the odds ratio of BILT classes (Table 5), there were no differences between parity 1 and 2, but the risk of having BILT above the threshold (> 4 μmol/L) in parity ≥3 was 3.85 times higher compared to party 1. This finding is in line with that of Walter et al. (25), who reported that the BILT baseline was higher in multiparous cows than in primiparous cows.

#### 3.1.5 Interaction between parity and DIM

The first order interaction between parity and DIM class was not significant for the traits investigated, except for cholesterol, globulin, and total protein. In particular, total protein and globulin were significantly different – i.e. Seventy nine.00 and 45.70 g/L - in parity >3 between 15 and 20 DIM compared with the rest of the interaction levels. Ferreira et al. (45) observed that total protein and globulin concentrations increased during lactation and in their study were greater in multiparous than in primiparous cows. Cholesterol, on the other hand, was the greatest in the last DIM class regardless of parity. Walter et al. (25) reported a constant increasing trend of cholesterol after calving and was greater in multiparous than in primiparous cows.

#### 3.1.6 Variation across seasons

Only few plasma traits were significantly affected by the effect of season, namely Na, Cl, K, albumin, globulin, urea, glucose and cholesterol. In this study, the sampling period spanned from May to October, excluding winter, late autumn, and early spring. In agreement with Cozzi et al. (15), the LSM estimated in this study for Na and Cl were the lowest in samples collected in July–August, while K concentration was the lowest in May–June (Table 6).

**Table 6:** Least squares means (standard error) of blood traits^1^ for the fixed effect of sampling season along with the *F*-value.

Blood trait	May–June	July–August	September–October	*F*-value
Energy profile				
Glucose, mmol/L	2.72^b^ (0.11)	2.83^b^ (0.11)	3.08^a^ (0.11)	8.02 ***
Cholesterol, mmol/L	2.83^a^ (0.16)	2.53^b^ (0.15)	2.80^ab^ (0.15)	4.17 *
NEFA, mmol/L	0.23 (0.04)	0.47 (0.07)	0.44 (0.07)	*ns*
BHB, mmol/L	0.67 (0.03)	0.63 (0.03)	0.51 (0.02)	*ns*
Urea, mmol/L	3.12^c^ (0.32)	3.61^b^ (0.30)	4.16^a^ (0.31)	10.45 ***
Mineral profile				
Na, mmol/L	140.02^a^ (0.70)	137.62^b^ (0.64)	139.68^a^ (0.67)	14.06 ***
K, mmol/L	4.22^b^ (0.08)	4.44^a^ (0.07)	4.50^a^ (0.07)	7.91 ***
Cl, mmol/L	98.93^a^ (0.68)	97.43^b^ (0.63)	99.88^a^ (0.65)	12.83 ***
Ca, mmoL/L	2.44 (0.02)	2.47 (0.02)	2.42 (0.02)	*ns*
P, mmol/L	1.63 (0.06)	1.60 (0.06)	1.56 (0.06)	*ns*
Mg, mmol/L	0.95 (0.01)	0.98 (0.01)	0.98 (0.02)	*ns*
Protein profile				
Total protein, g/L	74.38 (0.83)	74.07 (0.74)	73.29 (0.83)	*ns*
Albumin, g/L	31.47^c^ (0.66)	33.71^b^ (0.59)	35.93^a^ (0.64)	16.56 ***
Globulin, g/L	42.58^a^ (0.98)	39.78^b^ (0.87)	37.20^c^ (0.96)	10.18 ***
Hepatic and muscular profile				
AST, U/L	94.12 (3.90)	93.35 (4.06)	98.97 (4.17)	*ns*
GGT, U/L	16.86 (0.55)	15.81 (0.48)	14.15 (0.59)	*ns*
CK, U/L	229.14 (25.89)	186.38 (18.07)	263.00 (29.08)	*ns*
Stress profile				
Cortisol, nmol/L	9.81 (1.38)	14.85 (1.88)	19.51 (2.65)	*ns*

^1^NEFA, not esterified fatty acids (mmol/L); BHB, β-hydroxybutyrate (mmol/L); AST, aspartate aminotransferase (U/L); GGT, gamma glutamyl transferase (U/L); CK, creatin kinase (U/L). Estimates with different superscript letters within row are statistically significantly different (*p* ≤ 0.05).

With regard to the protein profile, both albumin and urea concentrations slightly increased during the sampling period, with the highest LSM estimated for September–October. In contrast, globulin concentration decreased within the time window considered (Table 6). Regarding the energy profile, the lowest concentration of glucose was found in May–June and July–August (2.72 and 2.83 mmol/L; Table 3) and samples collected in July–August had the lowest LSM for cholesterol concentration (2.53 mmol/L; Table 6). During the core of summer, in presence of acute heat stress, cows decrease their feed intake, inevitably reducing the energy available (46, 47). Moreover, the forage quality may differ across seasons in Italy. Although these two factors can be a reasonable explanation for the low cholesterol and glucose in July–August, this study lacks of data on individual feed intake, ration composition and diet protein level to support this hypothesis.

### 3.2 Fourier-transform mid-infrared spectroscopy

#### 3.2.1 Prediction ability of FT-MIR

The R^2^ and RMSE of the prediction models for plasma trait concentrations are shown in Table 7. The percentage of samples identified as outliers and excluded ranged from 0.60 to 4.60%, and the number of LV used ranged from a minimum of 2 to a maximum of 17.

**Table 7:** Goodness of fit statistics^1^ of the modified partial least squares regression models for blood traits^2^ developed using milk mid-infrared spectra.

Blood trait	% Outliers	n. LV	*R*^2^c	RMSEc	*R*^2^v	RMSEv
Energy profile						
Glucose, mmol/L	1.8	15	0.43	0.36	0.29	0.40
Cholesterol, mmol/L	2.1	9	0.47	0.64	0.37	0.70
NEFA, mmol/L	2.9	10	0.57	0.20	0.46	0.22
Log-NEFA	2.4	8	0.50	0.20	0.42	0.21
BHB, mmol/L	2.1	15	0.62	0.25	0.45	0.30
Log-BHB	1.2	15	0.62	0.13	0.52	0.15
Urea, mmol/L	1.8	17	0.89	0.43	0.86	0.48
Mineral profile						
Na, mmol/L	2.4	13	0.30	2.70	0.21	2.89
K, mmol/L	3.2	13	0.16	0.29	0.02	0.33
Cl, mmol/L	0.9	13	0.41	2.62	0.11	3.43
Ca, mmo/L	2.6	13	0.44	0.10	0.23	0.12
P, mmol/L	2.1	8	0.26	0.26	0.14	0.29
Mg, mmol/L	2.6	7	0.39	0.09	0.23	0.10
Protein profile						
Total protein, g/L	1.5	14	0.23	5.06	0.03	6.20
Albumin, g/L	1.8	9	0.38	3.14	0.32	3.64
Globulin, g/L	2.4	9	0.24	5.55	0.05	6.03
Hepatic and muscular profile						
AST, U/L	1.5	8	0.27	20.90	0.18	22.11
Log-AST	2.5	7	0.25	0.08	0.19	0.09
GGT, U/L	0.9	2	0.06	5.39	0.01	5.8
Log-GGT	1.8	4	0.08	0.14	0.02	0.16
CK, U/L	3.5	10	0.17	226.45	0.08	192.39
Log-CK	4.6	12	0.26	0.18	0.02	0.21
Stress profile						
Cortisol, nmol/L	3.0	9	0.25	10.28	0.08	12.63
Log-Cortisol	0.6	9	0.21	0.32	0.11	0.36

^1^LV, latent variables; *R*^2^_c_, coefficient of determination in calibration; RSME_C_, Root Mean Square Error in calibration; *R*^2^_V_, coefficient of determination in validation; RSME_V_, Root Mean Square Error in validation. The percentage of outliers (%) here refers to samples whose prediction was far from the actual value, i.e., large residual. ^2^NEFA, not esterified fatty acids (mmol/L); BHB, β-hydroxybutyrate (mmol/L); AST, aspartate aminotransferase (U/L); GGT, gamma glutamyl transferase (U/L); CK, creatin kinase (U/L).

Regarding the quality of predicted phenotypes, Williams (48) and Grelet et al. (49) proposed classifications based on the *R*^2^. In particular if the *R*^2^ is (i) ≥ 0.91, the model can be used for punctual prediction, (ii) between 0.82 and 0.90 the model is suitable for good quantitative screening; (iii) between 0.66 and 0.81, the model is adequate for approximate screening; and (iv) between 0.50 and 0.65, the predicted trait should be used exclusively for detecting extreme values or comparing groups (48).

**Figure 4:**
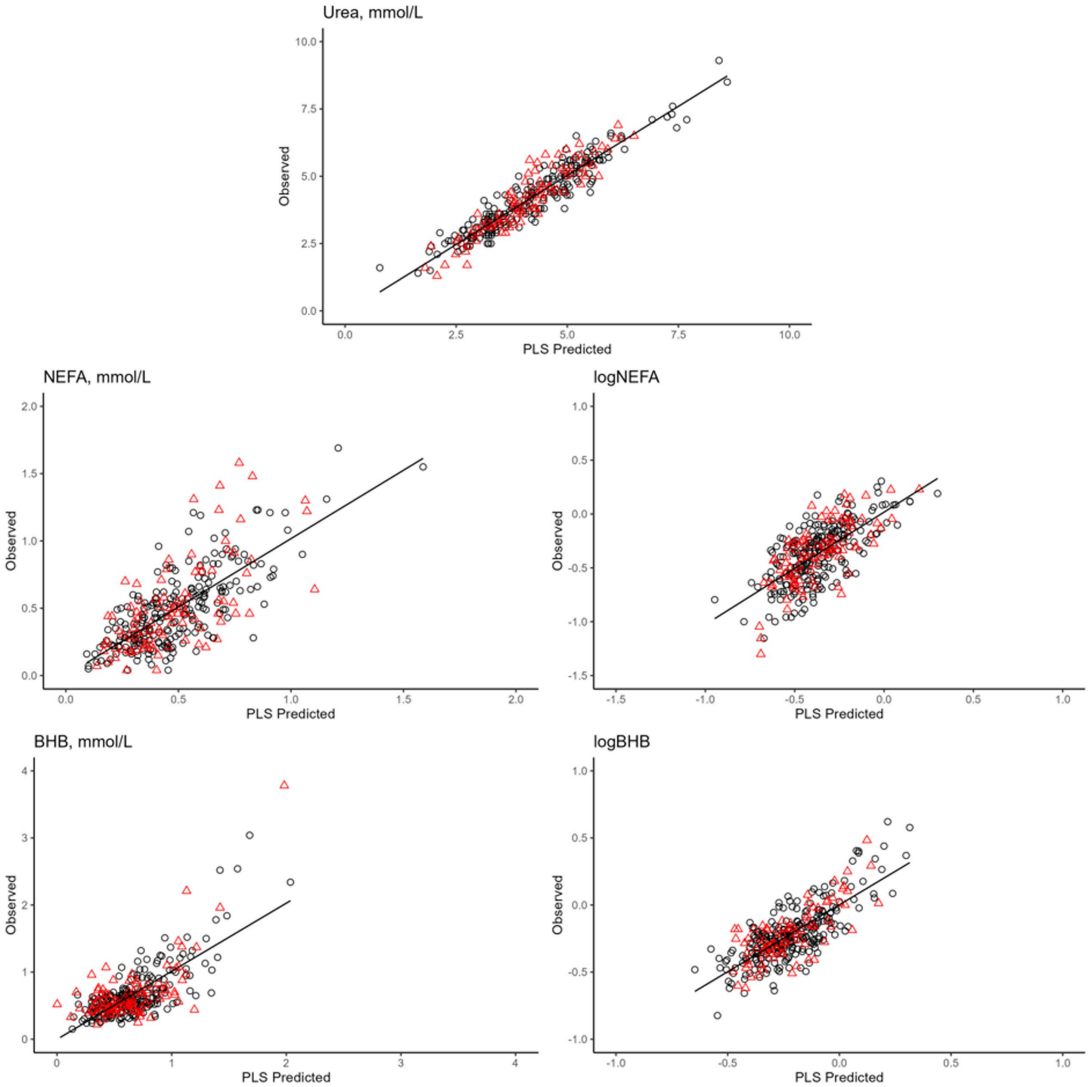
Plot of mid-infrared predictions (x-axis) and observed values (y-axis) for the blood traits^2^. Calibration set are reported in in red (▲), whereas validation set are black (●). ^1^Only prediction models with coefficient of determination in validation >0.40 are presented, namely: blood urea, not esterified fatty acids (NEFA), logarithmic transformation of NEFA (logNEFA), β-hydroxybutyrate (BHB), and logarithmic transformation of BHB (logBHB).

Overall, the prediction models developed for energy-related metabolites exhibited the best performance. In particular, the most outstanding model in terms of variance explained in the leave-one-out cross-validation was urea concentration (*R*^2^ = 0.89; Table 7; Figure 4). When applied to an external set, the same model still showed a very good performance (*R*^2^ = 0.86; Table 7; Figure 4). The prediction accuracy for blood urea in this study was higher than that reported in the literature (11, 50). In particular, Ho et al. (50), who used 3,027 samples of Holstein cows from 19 dairy herds in Australia to develop models, obtained R2 of 0.87 in cross-validation and 0.69 in external validation (50). On the other hand, Luke et al. (12) reported an *R*^2^ equal to 0.90 both in cross-validation and random validation on an independent external dataset. However, when validation was done using the herd-out approach (12), i.e., using an external independent farm, the performance reduced substantially (*R*^2^ = 0.35).

Prediction models for BHB concentration and log-transformed BHB had the same *R*^2^ in leave-one-out cross-validation (*R*^2^ = 0.62; Table 7; Figure 4), whereas in external validation it was slightly greater for the log-transformed trait (0.52 vs. 0.45; Table 7; Figure 4). Conversely, the prediction model for the untransformed NEFA concentration fitted better than that for the log-transformed NEFA in both calibration and validation. The *R*^2^ values in external validation were 0.57 and 0.46, respectively (Table 7; Figure 4).

Several studies have investigated the use of milk FT-MIR for predicting blood/serum metabolites, such as BHB and NEFA, to identify cows at risk of hyperketonemia, NEB, and excessive fat mobilization. The accuracy of NEFA and BHB predictions reported in this study are in line with those found previously by (11, 12, 50–52). Only Grelet et al. (52) found slightly greater *R*^2^ for BHB concentration both in calibration and validation, i.e., equal to 0.77 and 0.70, respectively. All these studies used blood sampled from cows in early lactation within 40 DIM (11, 12, 50, 52), while Giannuzzi et al. (51) investigated prediction models for these metabolites throughout the whole lactation, obtaining slightly lower accuracy for BHB prediction.

It is important to highlight that time is a key factor for the data quality and representativeness, with potential consequences in the model performance. When predicting a certain blood element from the milk spectrum, in fact, the distance – in time - between the blood and the milk sampling plays a crucial role (53). In fact, it has been demonstrated that the morning plasma level of NEFA is predicted with significantly higher accuracy when using evening (*R*^2^ = 0.61) than morning (*R*^2^ = 0.50) milk spectra (53). This indicates that, although the milk is still expected to mirror the blood, there could be a “delay” in some traits. In addition to this, there is evidence that several traits show very high variability during the day in healthy cows, with either peaks and/or long or short plateaus (e.g., glucose and cortisol).

Cholesterol and glucose were predicted with less accuracy than the traits mentioned above, with an R^2^ in leave-one-out cross-validation of 0.47 and 0.43, respectively (Table 7). These predictive abilities were generally similar to those reported by Benedet et al. (11), i.e., *R*^2^ equal to 0.39 and 0.20, respectively. Using the PLS, Giannuzzi et al. (51) obtained similar accuracy for cholesterol concentration, but, on the other hand, a greater accuracy for glucose concentration. To justify this difference, it is worth stressing that the study of Giannuzzi et al. (51) dealt with blood samples collected during the whole lactation, up to >305 DIM. As previously discussed, even if glucose tends to be high around calving, it may still vary quite substantially within the same day due to the presence of non-negligible peaks corresponding to meal administration or acute episodes of stress (28).

Blood mineral concentrations were generally predicted with less accuracy compared to the other traits. In fact, the R^2^ was below 0.50, regardless of the type of validation (Table 7) and the best model was the one developed for Ca concentration (*R*^2^ = 0.44), followed by Mg concentration (*R*^2^ = 0.39). When applied to an external set of samples, these two models showed lower R^2^ values (both equal to 0.23). Despite this, the results can be considered more promising compared to those reported by Luke et al. (12), who observed R^2^ in cross-validation of 0.08 and 0.06 for Ca and Mg, respectively. In their study (12), blood samples were taken immediately after or during milk sampling. Attempting to predict something present in the blood using the milk spectrum is challenging, as it is an indirect prediction, therefore low *R*^2^ are inevitably expected.

For the others plasma traits, the *R*^2^ in leave-one-out cross-validation was low, ranging from 0.08 (GGT) to 0.38 (albumin), as well as the one in external validation (0.01 to 0.32; Table 7). In general, whenever a trait was not normally distributed (e.g., BHB), prediction models were used for both the forms, log-transformed and untransformed. In general, the *R*^2^ values in the external validation were slightly higher when the log-transformed trait was used. In line with Luke et al. (12), the prediction model for albumin fitted better than globulin (Table 7). It is widely known that components present in very low concentrations are more difficult to predict with FT-MIR because of the small signal(s) within the spectrum. Highly concentrated components have strong absorption peaks and are thereby easier to be predicted precisely and accurately.

Based on the classification of Williams (48), concentrations of BHB, NEFA, and urea predicted with the model in Table 7 can be considered as good enough for population screening and for carrying out selective breeding. Phenotypes predicted from the milk spectra may be scarcely or moderately correlated with the real trait (e.g., blood BHB), but still the predictions can be valuable for genetic investigations at the population level and for design of breeding programs (54, 55). For example, FT-MIR allows to identify cows within a given population with high BHB and/or NEFA and their sires, i.e., bulls with progeny at major risk of metabolic diseases. The FT-MIR is a low-cost phenotyping tool since the analysis are regularly carried out for milk quality assessment in the framework of the official tests (14).

#### 3.2.2 FT-MIR classification ability

The PLS-DA was performed for traits whose threshold found a consensus in the literature, i.e., BHB, NEFA and BILT, considering two levels: equal to or below the threshold (no risk) and above the threshold (at risk). In the dataset 22.30, 8.90, and 27.80% of the samples were above the thresholds for BHB, NEFA and BILT, respectively (Table 8).

**Table 8:** Performance^1^ (%) of partial least squares discriminant analysis^2^ based on milk mid-infrared spectra in calibration and validation.

Trait	Threshold^3^	Calibration set					Validation set				
		Sensitivity	Specificity	TP	TN	Balanced accuracy	Sensitivity	Specificity	TP	TN	Balanced accuracy
BILT, μmol/L	4 μmol/L	0.48	0.96	0.80	0.84	0.72	0.42	0.91	0.78	0.68	0.67
BHB, mmol/L	1.2 mmol/L	0.52	0.99	0.96	0.85	0.76	0.33	0.98	0.94	0.60	0.66
NEFA, mmol/L	0.7 mmol/L	0.56	0.97	0.88	0.83	0.76	0.36	0.96	0.84	0.73	0.66

^1^TP, true positives correctly classified; TN, true negatives correctly classified. ^2^Binary classification (≤ vs. > threshold) for: NEFA, not esterified fatty acids (mmol/L); BHB, β-hydroxybutyrate (mmol/L); BILT, total bilirubin (μmol/L). ^3^Values refer to cut-offs retrieved from the literature [Ospina et al. (20)].

Sensitivity, defined for each plasma traits class as the probability of correctly identifying cows at risk (above the threshold), ranged from 0.48 for BILT to 0.56 for NEFA in calibration. In validation, the maximum and minimum sensitivity were 0.42 and 0.33, respectively (Table 8). Specificity, complementary to sensitivity, represents the probability of correctly identifying the control cases, i.e., cows not at risk, below the threshold. This parameter was close to unity, being overall ≥0.96 in calibration and ≥ 0.91 in validation. The sensitivity calculated in this study for BHB was 0.33 in validation, and not far from the value (0.28) reported by Benedet et al. (11). This percentage indicated that approximately one third of the cows at risk in this study were correctly identified. In the case of NEFA and BILT, this percentage was slightly higher: 36 and 42%, respectively. The fact that specificity is close to unity does not necessarily mean that the model robustness of this study is sufficiently good for implementation. In fact, the real aim in the field is to precisely identify potentially sick cows rather than those that are not. This means that sensitivity instead of specificity should be ideally improved and maximized. Martín-Gómez et al. (56) recommended an optimization based on sensitivity and specificity to assess the quality of PLS-DA models that discriminate between two classes. The balanced accuracy in calibration was equal to 0.72 for BILT and 0.76 for both BHB and NEFA.

Considering that health data and clinical events of dairy cows are not routinely recorded in Italy due to the absence of validated standardized protocols, binary phenotypes obtained through the PLS-DA could be valid indicator traits to be used for various purposes. However, further efforts should be made to improve the scalability and sensitivity of the models.

## 4 Conclusion

In the present study, we investigated non-genetic factors affecting the variability of blood traits in healthy Holstein cows in the early lactation and we attempted to predict them using milk FT-MIR. Most of the plasma traits were significantly affected by the fixed effect of parity, distance from calving, and season. The performance of the FT-MIR models – especially in classification – indicated that the use of milk spectra for monitoring hematic traits is advisable, especially for BHB, NEFA, and urea. In regression, however, our prediction models were not sufficiently accurate for a punctual determination of the concentration of the parameters. Despite of the mid to low accuracy, the predictions represent nowadays a valid opportunity for farmers and breeders for both decision-making and genetic screening at the population level, to better identify sick animals or cows at risk. Further efforts should be made to understand if the FT-MIR spectra – coupled with additional data from other sources, such as sensors and genomic information – analysed via alternative machine learning algorithms will result into better scalability, sensitivity, and accuracy.

## Data Availability

The datasets presented in this article are not readily available because the raw used in the present study belong to the Breeders Association of Veneto Region (ARAV, Vicenza, Italy), and were shared after agreement with the University of Padova. The raw data supporting the conclusions of this article will be made available from the corresponding author upon request.
